# Coercion Exercised upon Doctors in Peru

**Published:** 1867-06

**Authors:** 


COERCION EXERCISED UPON DOCTORS IN PERU.—The *Gaceta Medica* of Lima, contains the following enactment of the Governor of Arequipa. No medical man is allowed to refuse assistance to any one, either by night or day, under a fine of £10, which may be enforced by the party thus refused. The like penalty is incurred by any apothecary who shall refuse to make up a prescription or to administer any remedy, be it in the course of the night or day.—*London Lancet.*

